# An unusual presentation of takotsubo syndrome in an elderly patient

**DOI:** 10.1093/omcr/omad144

**Published:** 2024-02-16

**Authors:** Israr Khan, Adam Atoot

**Affiliations:** Department of Internal Medicine, Hackensack Meridian Health (HMH) Palisades Medical Center, North Bergen, NJ, USA; Insight Hospital and Medical Center Chicago, IL, USA; Department of Internal Medicine, Hackensack Meridian Health (HMH) Palisades Medical Center, North Bergen, NJ, USA

**Keywords:** Takotsubo syndrome, Apical ballooning syndrome, Broken heart syndrome, Stress cardiomyopathy, Acute coronary syndrome, Syncope, Transient ischemic attack

## Abstract

Takotsubo syndrome occurs predominantly in women and is usually preceded by acute emotional and/or physical stress. Patients commonly present with chest pain and/or dyspnea. Syncope or out-of-hospital cardiac arrest is a rare presentation. We report an unusual case of takotsubo syndrome in an elderly patient who presented with left facial droop, slurred speech, and syncopal episode. Initial presumed diagnosis was an ischemic stroke/transient ischemic attack. However, the patient was then treated for acute myocardial ischemia/infarction based on abnormal electrocardiogram, elevated cardiac troponin, and unremarkable neurological workup. Eventually diagnosed with takotsubo syndrome. Our case illustrates the importance of prompt identification particularly in the context of atypical presentation and further evaluation to rule out serious causes to mitigate related morbidity and mortality while simultaneously avoiding unnecessary investigations.

## INTRODUCTION

Takotsubo syndrome (TTS) was named after the Japanese octopus-trapping pot with a round bottom and narrow neck that resembles the shape of the left ventricle at the end of the systole on a ventriculogram. [1] Also described in the literature as ‘apical ballooning syndrome,’ ‘broken heart syndrome,’ and ‘stress cardiomyopathy’ [2]. It accounts for 1–3% of all cases of suspected acute myocardial infarction (AMI). [3] It occurs predominantly in women with a mean age of 58–75 years; however, cases in the literature have also been reported in younger women <50 and males—after physical exertion [1, 3]. Patients with TTS most commonly present with symptoms like chest pain 75.9% followed by dyspnea 46.9% [4]—that mimic acute coronary syndrome (ACS). Syncope or out-of-hospital cardiac arrest is a rare presentation [5].

Here in, we report an atypical presentation of takotsubo syndrome—as syncope, altered mental status, and facial droop.

## CASE REPORT

**Figure 1:**
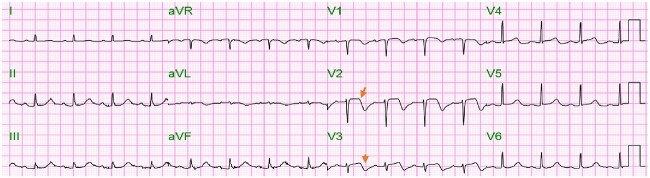
Electrocardiogram on arrival showed minimal ST-elevation with inverted T-wave inversion (* arrowheads*) in leads V2–V3.

**Figure 2:**
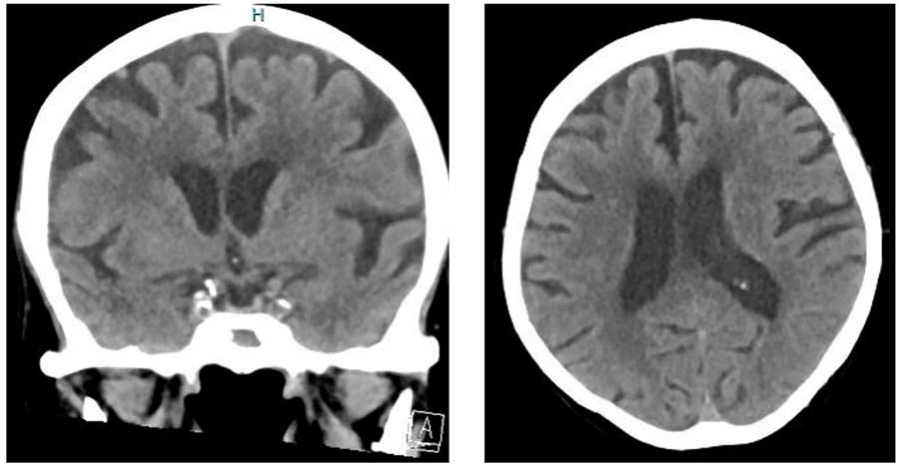
Computed tomography of the head demonstrated diffuse parenchymal volume loss and chronic microvascular changes but no acute intracranial pathology.

**Figure 3:**
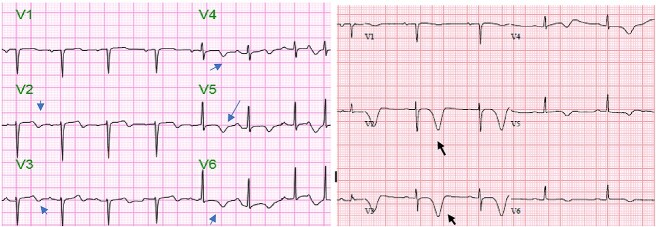
Follow-up electrocardiograms revealed resolution of ST-elevation with development of diffuse inverted T-wave (*blue arrows*) in precordial leads (Panel **A**) that evolved into deep symmetric T-wave inversions (black arrows) in V1–V2 (Panel **B**).

An 81-year-old female former smoker (60 pack-year) with a history of type 2 diabetes mellitus, cerebrovascular accident, and chronic obstructive pulmonary disease who presented to the emergency department with a history of left facial droop, slurred speech, and witnessed non-mechanical fall “several hours” prior to presentation with no reported head injury. Her home medications included atorvastatin, ticagrelor, and metformin. On arrival, patient was asymptomatic. Her temperature was 98.5°F, blood pressure was 88/52 mmHg, heart rate was 110/min, and oxygen saturation was 93% on room air. Physical exam was notable for altered level of consciousness (GCS score of 14 due to confusion on best verbal response). Initial cardiac troponin was 2.60 (reference range, <=0.05 ng/ml)] with creatinine level of 2.19 (reference range, 0.60–1.20 mg/dl). Other laboratory results are as shown in Table 1. Electrocardiogram (ECG) on arrival showed minimal ST elevation with inverted T waves in leads V2 and V3 shown in Fig. 1. Initial working diagnoses were stroke/transient ischemic attack, along with acute anteroseptal wall myocardial ischemia/infarction. A computed tomography scan of the head without contrast was done, which revealed no signs of acute intracranial pathology (Fig. 2). Patient received 325 mg of aspirin and started on intravenous unfractionated heparin. Cardiology and Neurology services were consulted. Repeat troponin level was 1.98 ng/ml, which had decreased from 2.60 ng/ml 1-h before. Serial ECGs showed resolution of transient ST elevation and development of bi-phasic inverted T-waves involving V1–V6 Fig. 3 (**Panel A**). Magnetic resonance imaging of the brain and magnetic resonance angiogram of head & neck were unremarkable. Transthoracic echocardiography (TTE) revealed mid-anteroseptal/apical akinesis with left ventricular ejection fraction (LVEF) of 40–45%—highly suggestive of takotsubo cardiomyopathy. A shared decision was made to postpone cardiac catheterization in the setting of acute kidney injury (AKI) and discontinue heparin drip. Meanwhile aspirin, atorvastatin, and ticagrelor were continued during hospitalization. The hospital course was complicated by heart failure with mild to moderate pulmonary edema and hypoxemia on day-6 due to fluid overload. On days 6–7, her biphasic T-wave evolved into deep symmetric T-wave inversion in V1–V3 as shown in Fig. 3 (**Panel B**)*.* A repeat TTE on day 10—showed improvement in LVEF (65–70%) along with normal LV segmental wall motion. Her heart failure resolved, and her creatinine had improved. Her abnormal T waves persisted but prolonged QT resolved on follow-up ECG. The patient was discharged to the cardiac rehab center after 3 weeks of hospitalization on aspirin, atorvastatin, and low-dose beta-blocker.

**Table 1:** Blood work-up on admission and discharge

CBC (Complete blood count)			
Parameters	Results		Normal values (range)
	On admission	On discharge	
White blood cells (WBC)	8.7	7.8	4000–11 000 cell/ul
Hemoglobin	12.5	9.1	12.0–15.5 g/dl
Hematocrit	38.3	26.8	36–46%
Mean corpuscular volume	95	92.9	81–99 Fl
Red cell distribution width	15	15.3	11.5–14.5%
Platelet count	300 × 10^3^	462	150–400 × 10^3^
CMP (Comprehensive metabolic panel)			
Glucose	155	109	70–105 mg/dl
Blood urea nitrogen (BUN)	44	30	7–25 mg/dl
Creatinine	2.19	1.20	0.60–1.20 mg/dl
EGFR, Non-African American	21.54	44	>60 (ml/min/1.73 m^2^)
Sodium	139	141	136–145 mmol/l
Potassium	4.0	4.9	3.5–5.00 mmol/l
Chloride	101	103	96–108 mmol/l
Calcium	8.8	9.5	8.6–10.5 mg/dl
Magnesium	2.10	1.8	1.9–2.7 mg/dl
Chemistry-Other			
Troponin	2.09➔1.98➔2.60➔0.14	0.04	≤0.05 ng/ml
Thyroid stimulating hormone	0.50	↔	0.45–5.3 uIU/ml

EGFR: Estimated glomerular filtration rate, ESR: Erythrocyte sedimentation rate, GCA: Giant cell arteritis

## DISCUSSION

According to literature, the most common presentation of patients with TTS is chest pain and/or dyspnea mimicking ACS, however, our case presented with syncope, left facial droop, and altered mentation. There have been some reported cases of Takotsubo syndrome in the literature with unusual presentation. Arunachalam *et al.* [6] reported 83-year-old female with extreme thirst as presenting complaint, troponin level of 4.241 ng/ml, T-wave inversions in the inferior leads on ECG, and LVEF of 30%. Clark *et al.* [7] reported a 68-year-old female with acute dyspnea due to status asthmaticus, mildly elevated troponin level with ECG findings of ST segment elevations. Muratsu *et al.* [8] reported two hemodialysis dependent patients. Case 1 was a 63-year-old female who had generalized tonic seizures. ECG revealed inverted T waves and QT prolongation in all leads, with negative troponin t test. Case 2 was a 59-year-old female who presented with fatigue and had giant inverted T waves in leads V1–V5 on routine ECG, with normal troponin level.

Most common complication is mild to moderate heart failure with or without pulmonary edema. Coronary angiography (CAG) with left ventriculography is considered gold standard to rule in or rule out TTS due to the lack of reliable non-invasive diagnostic tools [9, 10]. To rule out diagnoses, modalities such as cardiac MRI and CCTA are both reliable options. For patients who are at high-risk of complications from CAG, CCTA is recommended. However, for the sub-acute phase, cardiac MRI with late gadolinium enhancement is more helpful, according to Ghadri *et al*. (Part 2), [10] International Takotsubo Diagnostic Criteria were proposed in 2018 for diagnosing TTS based on International Consensus (Table 2) [10]—differ from the widely known Revised Mayo Clinic Criteria due to the absence of obstructive CAD or angiographic evidence of acute plaque rupture; pheochromocytoma, myocarditis, recent significant head trauma, intracranial bleeding, and hypertrophic cardiomyopathy (HOCM) [9]. The differential diagnoses in regard to our case were ischemic stroke/TIA, anteroseptal wall MI, Wallen’s syndrome, TTS, and arrhythmogenic right ventricular cardiomyopathy. Nevertheless, a diagnosis of TTS was made given the patient’s postmenopausal age, atypical symptoms, new ECG findings, TIA, absence of neurological bleeding, slight elevation in troponin level, mid-anteroseptal/apical akinesis on TTE, improvement of LVEF and resolution of wall motion abnormalities within few weeks.

**Table 2:** International Takotsubo Diagnostic Criteria (2018)

1) Transient hypokinesis, akinesis, or dyskinesis in the left ventricular presenting as apical ballooning or midventricular, basal, or focal wall motion abnormalities; regional wall motion abnormalities that extend beyond a single epicardial vascular distribution. 2) Preceding of an emotional, physical, or combined trigger, but not always present. 3) New ECG abnormalities (ST-segment elevation, ST-segment depression, T-wave inversion, and QTc prolongation) or modest elevation in cardiac troponin. 4) Neurologic disorders (e.g. subarachnoid hemorrhage, stroke/transient ischemic attack, or seizures), as well as pheochromocytoma, may serve as triggers. 5) Levels of cardiac biomarkers (troponin and creatine kinase) are moderately elevated with significant elevation of brain natriuretic peptide is common. 6) Significant coronary artery disease (CAD) is not a contradiction. 7) Patients have no evidence of infectious myocarditis. 8) Postmenopausal women are primarily affected population.

Treatment is usually supportive care—as there is no established therapy and symptoms resolve within a few weeks. However, initial management is the same as AMI with aspirin, intravenous heparin, and morphine and oxygen if needed, beta-blockers unless contraindicated, serial ECGs, and troponins due to similar presentation [10]. Prognosis is usually favorable. Rate of stroke/TIA was reported to be higher (1.7% per patient year) than MI in patients with TTS. The reported recurrence rate was 1.8% per patient-year with a duration range from 25 days to 9.2 years after the first incident. [4]

Takotsubo syndrome mimics ACS, making it challenging to distinguish between ACS and takotsubo syndrome based on electrocardiograms and cardiac troponins, especially in cases of atypical presentation. Early cardiac catheterization is an effective way to rule out serious causes, reduce morbidity and mortality, and avoid misdiagnosis in cases where TTS may be mistaken for ACS; thereby, simultaneously avoiding unnecessary investigations.

## CONFLICT OF INTEREST STATEMENT

No conflict of interest.

## FUNDING

The authors have not declared a specific grant for this research from any funding agency in the public, commercial or not-for-profit sectors.

## ETHICAL APPROVAL

None required.

## CONSENT

Obtained from next of kin and surrogate decision maker, as patient was not fully oriented.

## GUARANTOR

Israr Khan.
